# The Contribution of Drugs and* Helicobacter pylori* to Gastric Mucosa Changes in Patients with Systemic Lupus Erythematosus and Antiphospholipid Syndrome

**DOI:** 10.1155/2019/9698086

**Published:** 2019-05-05

**Authors:** Tatiana M. Reshetnyak, Irina A. Doroshkevich, Natalia V. Seredavkina, Evgeny L. Nasonov, Igor V. Maev, Vasiliy I. Reshetnyak

**Affiliations:** ^1^Department of Vascular Rheumatology, VA Nasonova Research Institute of Rheumatology, Kashirskoe shosse, 34A, 115522, Moscow, Russia; ^2^Department of Rheumatology, Russian Medical Academy of Postgraduate Education, Barrikadnaya str., 2/1, 125993, Moscow, Russia; ^3^Municipal Outpatient Clinic No 36, Moscow Department of Health, Novomar'inskaya str., 2, 109652, Moscow, Russia; ^4^Department of Systemic Connective Tissue Diseases, VA Nasonova Research Institute of Rheumatology, Kashirskoe shosse, 34A, 115522, Moscow, Russia; ^5^Department of Propaedeutic of Internal Diseases and Gastroenterology, A.I. Yevdokimov Moscow State University of Medicine and Dentistry, Delegatskaya St., 20, p. 1, 127473, Moscow, Russia

## Abstract

**Background:**

The nature and rate of gastric mucosal (GM) damage in systemic lupus erythematosus (SLE) and antiphospholipid syndrome (APS) remain to be among the unsolved problems.

**Objective:**

To define the role of* H. pylori* and drugs in the development of GM damages in SLE and APS.

**Methods:**

A study was conducted on 85 patients with SLE and APS. All the patients underwent esophagogastroduodenoscopy with targeted biopsy of the mucosa of the gastric body and antrum. The presence of* H. pylori* in the gastric biopsy specimens was determined using polymerase chain reaction.

**Results:**

Endoscopic examination revealed that the patients with SLE and APS on admission had the following GM changes: antral gastritis (82.4%), erosions (24.7%), hemorrhages (8.2%), and pangastritis (8.2%). SLE and APS patients showed no direct correlation between the found GM damages and the presence of* H. pylori*. The use of glucocorticoid, low-dose acetylsalicylic acid, nonsteroidal anti-inflammatory drug, and anticoagulant in SLE and APS patients is accompanied by GM damage.

**Conclusion:**

There was no evidence of the role of* H. pylori* in GM damage in the SLE and APS patients. More frequent detection of* H. pylori* was observed in anticoagulants or low-dose acetylsalicylic acid users than in glucocorticoids and nonsteroidal anti-inflammatory drugs ones.

## 1. Introduction

Systemic lupus erythematosus (SLE) is a chronic systemic autoimmune disease characterized by exacerbations and remissions, as well as by diverse clinical manifestations that can range from minor signs, such as fatigue, weight loss, and arthralgia, to life-threatening damage to the kidneys or central nervous system [1–3]. The involvement of the digestive system in the pathological process and its damage remains an insufficiently studied problem in SLE. The clinical manifestations of these lesions in SLE are variable and are associated with the involvement of any of digestive system segments [4–6]. There is no clear idea: whether the clinical signs of digestive system damage are a sequel of SLE, or whether they are nonspecific and associated with infection, thrombosis, and medication [7–9]. The study of this issue in SLE is of great importance since digestive system damage can affect the course of the disease [10–12]. The description of the antiphospholipid syndrome (APS) initially within the framework of SLE and then as a primary acquired thrombophilia led to the identification of new clinical manifestations associated with ischemia of various organs, including the organs of the digestive system [13–17]. The nature and rate of gastric mucosal (GM) damage in APS remain to be among the unsolved problems. The detected GM damages in patients with SLE and APS may depend on the activity of disease, on the presence of* Helicobacter pylori (H. pylori*), and on the impact of therapy [5, 10, 14, 18–20]

The discovery of the spiral-form bacterium* H. pylori* in 1983 has drawn attention to the study of their role in the etiopathogenesis of gastroduodenal diseases [21]. It has been suggested that there is a relationship between* H. pylori* infection and autoimmune and hematological disorders in systemic rheumatic diseases [22, 23]. In addition, whether the viruses of the* Herpesviridae* family can be involved in the development of active gastritis, GM ulcerous defects, and hemorrhages, particularly in patients on immunosuppressive therapy or in those with immunodeficiency, is being actively discussed now [24–28].

Glucocorticoids (GCs) are the drugs of choice for SLE. A number of authors believe that the risk for gastrointestinal complications during therapy with GCs is low and is associated with their total dose, the duration of therapy, and the simultaneous use of nonsteroidal anti-inflammatory drugs (NSAIDs), including aspirin (acetylsalicylic acid) [20, 29, 30]. The causes and nature of GM changes in these patients continue to be debated [5, 8–10, 26].

*The objective* of this study was to define the role of* H. pylori,* the viruses of the* Herpesviridae* family, and drugs in the development of GM damage in patients with systemic lupus erythematosus and antiphospholipid syndrome.

## 2. Patient Characteristics and Methods

A prospective study was conducted on 85 patients with SLE and APS. The research is based on the study of the detection of* H. pylori, HSV-1, *and* CMV* and changes in the gastric mucosa of these patients at the time of inclusion to the study. None of the patients received anti-Helicobacter and antiviral therapy at the time of inclusion in the study and last six months before. Sixty-five patients were diagnosed as having SLE according to the 1997 ACR diagnostic criteria [31]. Twenty-six of the 65 SLE patients were detected to have reliable APS (the 2006 criteria) [32]. Of the 85 examined patients, 20 cases had verified primary APS (PAPS) [33]. The study inclusion criteria were the accurate diagnosis of SLE, APS, and PAPS and a patient's signed informed consent. The patients' age at the time of examination ranged from 15 to 68 years (mean age, 36.7 ± 13.1 years) and the disease duration was 6 months to 33 years (mean disease duration, 11.6 ± 5.6 years). According to the diagnosis, the patients were divided into 3 groups, the characteristics of which are presented in Table 1.

All the patients included in the study were interviewed using a specially designed schedule that included gastrointestinal complaints (for the entire period of the disease and at the time of study inclusion) and risk factors for GM changes; during the same periods, drug therapy was also recorded. SLE activity was assessed in SLEDAI scores [34].

Forty-nine Patients took anticoagulants (ACs).

Twelve (30.8%) of the 39 SLE patients without APS had taken neither GCs nor LDASA nor NSAID nor anticoagulants (ACs) for 6 months or more before the study inclusion. The main clinical manifestations of SLE in these patients were associated with skin and mucosal lesions accompanied by immunological disorders that needed no treatment. These patients formed a comparison group.

The study exclusion criteria were Zollinger-Ellison syndrome, grades 2-3 circulatory failure, grades 2-3 respiratory failure, chronic renal failure, and liver cirrhosis.

All the patients underwent esophagogastroduodenoscopy (EGD) using an Olympus XP-20 (Japan) with targeted biopsy of the mucosa of the gastric body and antrum.

### 2.1. Laboratory Studies

A polymerase chain reaction (PCR) was used to detect* H. pylori* in gastric biopsy specimens.* H. pylori, HSV-1, *and* CMV* DNA was isolated and detected in the gastric biopsy specimens in accordance with the procedure and instructions for the PCR test-systems DiaGen-*Helicobacter*®, DiaGen-*CMV*®, and DiaGen-*HSV*® (ZAO “Laboratory Genetic Engineering Systems (LAGES), Moscow, Russia). UreC- and CagA (cytotoxin-associated gene A)-targeting primers (DiaGen-*Helicobacter*®) were employed to detect* H. pylori* DNA. Primers for* CMV* and* HSV-1* DNA contained the species-specific segments of the respective viral polymerases (DiaGen-*CMV*® and DiaGen-*HSV*®).

### 2.2. Statistical Analysis

The results obtained were statistically processed using the nonparametric methods of the statistical program “VassarStats: Website for Statistical Computation”, “Statistics”, and “EpiInfo”. The Mann-Whitney test was carried out to compare quantitative indicators. The statistical significance of the indicators was determined at P<0.05. The qualitative indicators in 2 unrelated groups were compared in the 2x2 contingency table using the *χ*^2^ test; Fisher's exact test was applied if the number of observations was less than 5.

## 3. Results

### 3.1. Clinical Manifestations, the Detection Rate of* H. pylori*, and Viruses of the* Herpesviridae* Family in the Examined Patients

The clinical symptom complex indicating gastric pathological changes in patients with SLE and APS included pain syndrome and manifestations of gastric dyspepsia. Medical history data showed that epigastria pain was observed in 61 (71.8%) of the 85 patients. The survey of the patients indicated that epigastria pain was recorded significantly (*χ*^2^ = 3.65; OR = 2.50; 95% CI, 1.11-10.10; P = 0.05) more frequently in patients with APS (38/46) compared to those with SLE without APS (23/39). Forty-two (49%) patients had a past history of heartburn; nausea occurred periodically in 23 (27%) patients.

The signs of SLE activity and concomitant drug therapy may affect the appearance of the symptom complex of gastric dyspepsia. At the time of study inclusion, the activity of SLE in 8 (12.3%) patients was manifested by glomerulonephritis and in 12 (18.4%) by central nervous system lesions out of 65 patients with SLE. The Systemic Lupus Erythematosus Disease Activity Index (SLEDAI) score was 17 ± 4 in patients with signs of gastrointestinal dyspepsia and was comparable with that (16 ± 6) in those without these signs (P > 0.05).

Table 2 gives data on the detection of the examined infectious agents in the patient groups. Infectious agents, such as* H. pylori*,* HSV-1*, and* CMV*, were identified in the gastric biopsy specimens of 75 (88.2%) of the 85 examined patients.

The detection rate of* H. pylori* in the gastric biopsy specimens of the examined patients was high and ranged from 70% to 81% in the groups. A PCR assay revealed* H. pylori* in 88.0% of the 75 patients with GM infection.* H. pylori CagA*-positive strains were present in 71.2% of the 66 patients with* H. pylori*. The* H. pylori CagA*+ strains exhibited a tendency to be more frequently detected in SLE patients without APS; but these differences were not statistically significant. In the entire group of the examined patients, the detection rate of* H. pylori* in the GM biopsy specimens was significantly higher than that of* HSV-1 *and* CMV *(Table 2).

### 3.2. GM Changes at Endoscopy and Detection Rate of* H. pylori* and Viruses of the* Herpesviridae* Family in the Examined Patients

The EGD findings suggested that 77 (90.6%) of the 85 patients had GM changes. At the time of examination, 48 (62.3%) of the 77 patients had no complaints. Endoscopic examination revealed that the 85 examined patients with SLE and APS had the following GM changes: antral gastritis (n = 70 (82.4%)), pangastritis (n = 7 (8.2%)), erosion (n = 21 (24.7%)), and hemorrhage (n = 7 (8.2%)). Pangastritis, erosion, and hemorrhage were concurrent with antral gastritis. The intact GM was present in 8 (9.4%) of the 85 examined patients.

EGD study most frequently revealed antral gastritis in the examined patients. Antral gastritis, erosions, hemorrhages, and intact gastric mucosa were found in both patients with identified infectious agents and those without them. It should be noted that infection was identified in 75% of the 8 patients with intact GM.

PCR detection rates of infectious agents in different GM changes at endoscopic examination are given in Table 3.

*H. pylori* was dominant among the identified infectious agents associated with GM changes detected at EGD. The presence of* H. pylori *was combined with* Herpesviridae *in 36 (48.0%) of 75 patients with infected GM. Thirty-nine (52.0%) of the 75 patients were found to have monoinfection: with* H. pylori* (n = 29),* CMV* (n = 3), and* HSV-1* (n = 7).

Among the 70 patients with antral gastritis, there were 77.1%* H. pylori*-positive patients.* H. pylori* was detected in all the patients with pangastritis. Four of the 7 patients with pangastritis had* H. pylori CagA*-positive strains.

GM erosions were found in 21 patients and were concurrent with antral gastritis in all cases.* H. pylori* was detected in 81.0% of the patients with GM erosive changes.* H. pylori CagA*-positive strains were present in 7 (41.2%) of the 17 patients.

*H. pylori* was detected in 5 of the 7 patients with GM hemorrhages. Hemorrhages were concurrent with other manifestations of GM damages: with pangastritis in 2 cases, with antral gastritis in 3 cases, and simultaneously with antral gastritis and erosions in 2.

Neither* H. pylori* nor* CMV* and* HSV-1* were detected in 2 (25%) of the 8 patients with intact GM.

Table 4 presents endoscopic GM changes in the examined groups according to the presence or absence of* H. pylori*.

Antral gastritis was detected in all the patients in the group of patients with APS (PAPS and SLE+APS) without* H. pylori*. At the same time, antral gastritis was present in only 78.6% of patients with PAPS and* H. pylori*-positive. The patients with SLE showed a reverse pattern: GM injures were seen in 90.3% of the H. pylori-positive cases and in 62.5% of the H. pylori-negative cases (P > 0.05), (Table 4).

*HSV-1* and* CMV* were detected in the gastric biopsy specimens from 10 (14.3%) of the 70 patients with antral gastritis. Out of them, 4 (40.0%) had antral gastritis concurrent with GM erosions and hemorrhages.

Five (71.4%) of the 7 patients with pangastritis had gastrointestinal complaints. Isolated strain* H. pylori* positive for CagA was detected in the 6 patients. Pangastritis was revealed in one PAPS patient positive for* H. pylori* and cytotoxicity of* CagA*. This patient had epigastric pain and dyspeptic complaints. In the SLE+APS group, pangastritis concurrent with GM hemorrhages was noted in one patient positive for* H. pylori*. The patient had no digestive complaints at the time of examination. In one (20.0%) of the 5 patients with SLE without APS, pangastritis was concurrent with GM hemorrhages. Two out of five patients with SLE without APS had pangastritis and complained of heartburn at the time of examination

GM erosions were identified in 27.3% of the 77 patients with GM changes. GM erosions tended to more frequently develop in patients with APS (15 out of the 46 patients with PAPS and SLE+APS) compared to those with SLE (6 out of the 39 patients with SLE) (P = 0.056; OR = 2.66; 95% CI, 0.92-7.73; RR = 2.12, 95% CI, 0.91-4.94).

Multiple GM erosions were noted to be significantly frequently detected in patients with APS compared to those with SLE: in 12 of the 15 patients with APS, whereas multiple erosions were present in 1 of the 6 patients in the SLE group* (P = 0.0067*; OR = 24; 95% CI, 2.04-282.69; RR = 5.6; 95% CI, 0.90-34.99). Multiple GM erosions were concurrent with acute gastric ulcer and* H. pylori CagA*-positivity in one patient with SLE+APS.

The two patients with PAPS and GM hemorrhages were found to have hepatic vein thrombosis (Budd-Chiari syndrome). In the patients with Budd-Chiari syndrome, portal hypertension had no signs of bleeding from the esophageal veins. GM hemorrhages were concurrent with multiple GM erosions in two PAPS patients with Budd-Chiari syndrome. One of these patients was* H. pylori*-positive and the other was* H. pylori*-negative. Among the SLE patients without APS, GM hemorrhages were associated with the presence of* H. pylori*-positive and concurrent with pangastritis in both cases.

Five (62.5%) of the 8 patients with intact GM were observed to be positive for* H. pylori* (Table 4).

## 4. Endoscopic GM Changes in Relation to* H. pylori* Detection and Therapy with Glucocorticoids, Aspirin, Nonsteroidal Anti-Inflammatory Drugs, and Anticoagulants in the Examined Patients

At the time of study inclusion, 73 (85.9%) of the 85 patients received different drugs: GCs, LDASA, NSAIDs, and ACs, as clinically indicated. Sixty-nine (94.5%) of the 73 patients taking GCs, NSAIDs, LDASA, or ACs had GM changes and only 4 (5.5%) patients had intact GM.

Twelve (14.1%) of the 85 patients had not taken GCs, NSAIDs, LDASA, or ACs within 6 months or more prior to study inclusion. GM changes were detected in 8 (66.7%) of the 12 patients; 4 (33.3%) had intact GM. The patients who had not received GCs, NSAIDs, LDASA, or ACs within 6 months or more at the time of study inclusion served as a comparison group.

GM injuries were significantly more frequently detected in the group of patients taking GCs, NSAID, LDASA, or ACs (69/73) versus 12 patients in the comparison group (8/12) (*P = 0.012*; OR = 8.63; 95% CI, 1.80- 41.35).

GM changes were significantly more commonly observed when comparing 12 patients in the comparison group (8/12) with a specific group of patients receiving GCs, ACs, LDASA, LDASA+NSAIDs (Table 5).

Comparison of the number of patients with and without GM changes revealed no significant differences between the patient groups by the presence or absence of* H. pylori* in those who took a certain drug (P_2_, Table 5). Comparing the number of patients with altered GM showed no significant differences in the presence or absence of* H. pylori* between the comparison group and the group taking the appropriate drug.

*H. pylori* was significantly more frequently detected in the patients with altered GM who took LDASA (33* H. pylori*-positive cases and 4* H. pylori*-negative cases;* P = 0.031*; OR = 3.58; 95% CI, 1.05–12.17) and ACs (41* H. pylori*-positive cases and 6* H. pylori*-negative cases;* P = 0.038*; OR = 2.96; 95% CI, 1.01-8.68) compared to those who received GCs (30* H. pylori*-positive cases and 13* H. pylori*-negative cases). At the same time, none of these patients received anti-*Helicobacter* therapy at the moment of examination.

The number of patients with GM changes and* H. pylori* tended to increase in patients who took LDASA (33/37) compared to those who received LDASA+NSAID (13/19) and had* H. pylori* too (P = 0.06).

Table 6 gives data on the nature of GM changes at EGD in SLE patients (n = 65) in the GC, non-GC, and comparison groups, as well as their association with the presence or absence of* H. pylori*. Of the 85 cases, 41 (48.2%) patients (18 with SLE, 3 with SLE and APS, and 20 with PAPS) did not took GCs at the time of study inclusion. Seven of the 41 patients had previously received CGs but discontinued for various reasons within the past one year or more before the study inclusion. Forty-four (73.3%) of the 65 patients with SLE and SLE+APS had used GCs for 5.6 ± 7.2 (1 to 33) years.

The GM was intact in only one of the 44 patients using GCs. GM changes were significantly more common in the GC group (43/44) than in the non-GC one (16/21) (*P = 0.011*; OR = 13.44; 95% CI, 1.46-124.03) and in the comparison group (8/12) (*P = 0.006*; OR = 21.5; 95% CI, 2.12-218.27). There were no significant differences in GM changes between the non-GC and comparison groups.

There were no significant differences in the detection rate of* H. pylori* in the comparison and GC groups (Table 5). There were no significant differences also in patients with appropriate GM changes according to the presence or absence of* H. pylori* between the GC and non-GC groups (*P*_*2*_, Table 6) and when analyzing with the comparison group (*P*_*4*_, Table 6).

The detection rate of GM erosions in the GC group (27.3%) was higher than in the non-GC (19.1%) and comparison (16.7%) groups. At the same time, GM erosions in patients receiving and not receiving GCs were accompanied by* H. pylori* infection in 75% of cases.

According to the standards for thrombosis prevention and treatment, ACs are pathogenetically justified for APS patients.  Table 7 gives data on the nature of GM changes at EGD in the anticoagulant (AC), non-AC, and comparison groups, as well as their association with the presence or absence of* H. pylori*.

Out of the 85 patients, 49 (57.6%) cases, including 46 patients with APS (20 with PAPS and 26 with SLE+APS) and three with SLE took ACs at the time of study inclusion. Thirty-six patients with SLE did not receive ACs. Of the 49 patients, 16 (with PAPS (n = 10), SLE+APS (n = 3), and SLE (n = 3)) received low-molecular-weight heparins (LMWH). Thirteen of the 16 patients used vitamin K antagonists (warfarin) prior to LMWH administration. The mean duration of LMWH therapy did not exceed 30 days in these patients who then were again switched to warfarin. Three patients with SLE took LMWH for the prevention of thrombosis due to hypercoagulation because of lupus glomerulonephritis. At the time of study inclusion, 33 patients with PAPS and SLE+APS received warfarin.

GM changes were significantly more frequently seen in the AC group (47/49) than in the comparison group (4/12) (*P*_*1*_* = 0.011*). There were no significant differences in the detection rate of GM changes in the non-AC and comparison groups and between the AC and non-AC groups.

GM* H. pylori* in the AC group was detected in 42 (85.7%) of the 49 patients, which was significantly more common than in the comparison group (7/12) (*P = 0.047*; OR = 4.29; 95% CI, 1.06-17.36) and in the non-AC group (20/36) (*P = 0.002*; OR = 4.8; 95% CI, 1.70-13.52). There were no differences in the detection rate of GM* H. pylori* in the comparison (7/12) and non-AC (20/36) groups.

According to the presence or absence of* H. pylori*, there were no significant differences between the AC, non-AC, and comparison groups in patients having appropriate GM changes (*P*_*2*_ and* P*_*4*_ in Table 7).

The AC group showed a significant rise in the number of antral gastritis patients (45/49) versus the comparison group (5/12) (*P*_*1*_* = 0.0004*) and the non-AC (22/36) one (*P*_*3*_* = 0.00076*) (Table 7).

The frequency of other GM changes in the AC, non-AC, and comparison groups was statistically insignificant. At the same time, GM changes in all the patient groups were observed with the high rate of* H. pylori* infection (Table 7).

In the AC and non-AC groups, GM erosions were noted in 22.5 and 27.8% of cases, respectively, which was more frequent than in the comparison group (16.7%). There were no significant differences in GM changes between the non-AC and comparison groups.

LDASA and NSAID are commonly prescribed for treating SLE and APS patients. Sixty (70.6%) of the 85 examined patients took LDASA (50-100 mg/day) and NSAID with LDASA (LDASA+NSAID): 46 patients diagnosed with APS and 14 with SLE without APS. Forty of the 60 patients used only LDASA without NSAIDs. Twenty of the 60 patients also received irregularly NSAID simultaneously with LDASA. The mean duration of NSAID intake ranged from 6 months to 3 years. Twenty-five of the 85 patients received neither LDASA nor LDASA+NSAID at the time of examination. These patients and the comparison group were used to analyze the effect of LDASA on the GM status.

Table 8 presents data on the nature of GM changes at endoscopy in the LDASA-treated patients (n = 40), who did not take LDASA+NSAID (n = 25) and in the comparison group (n = 12), as well as their association with the presence or absence of* H. pylori*.

In the LDASA group, intact GM was detected in 5 (12.5%) of the 40 patients, which was more frequent than when using GCs (2.3%), ACs (4.1%), or LDASA+NSAID (5.0%).

GM changes in the LDASA group (37/40) were significantly more common than those in the comparison group (8/12) (*P = 0.041*; OR = 6.17; 95% CI, 1.15-33.11). There were no significant differences in the detection rate of GM changes between the non-LDASA+NSAID and comparison groups, as well as between the LDASA and non-LDASA+NSAID groups.

In the LDASA group, GM* H. pylori* was detected in 35 (87.5%) of the 40 patients, which was significantly more common than in the comparison group (7/12) (*P = 0.038*; OR = 5; 95% CI, 1.14-22.00) and in the non-NSAID+LDASA group (16/25) (*P = 0.028*; OR = 3.94; 95% CI, 1.14-13.65). There were no differences in the detection rate of GM* H. pylori* in the comparison (7/12) and non-NSAID+LDASA (16/25) groups. There were no significant differences also in the detection of* H. pylori* in the presence of various GM changes between the LDASA, non-LDASA+NSAID, and comparison groups (*P*_*2*_, and* P*_*4*_ in Table 8).

There was a significant increase in the number of patients with antral gastritis in the LDASA group (32/40) versus the comparison one (5/12) (*P*_*1*_* = 0.016*). There were no significant differences in the detection rate of antral gastritis in the LDASA and non-LDASA+NSAID groups (Table 8). Antral gastritis also prevailed in the non-LDASA+NSAID group (20/25) versus the comparison one (5/12) (*P = 0.026*; OR=5.6; 95% CI 1.24-25.33).

Pangastritis was unassociated with the administration of LDASA. There was no case of pangastritis in patients receiving LDASA without NSAID, which significantly differed in its incidence in the comparison group (2/12) (*P*_*1*_* = 0.05*) and in the non-LDASA+NSAID one (5/25) (*P*_*3*_* = 0.006*). The frequency of other GM changes was statistically insignificant in the LDASA, non-LDASA+NSAID, and comparison groups (Table 8).

Twenty (33.3%) of the 60 patients receiving LDASA took NSAIDs irregularly. Among them, there were 10 (25.6%) patients with SLE without APS, 9 (34.6%) with SLE+APS and one (5.0%) with PAPS.  Table 9 gives data on the nature of GM changes at endoscopy in the NSAID+LDASA (n = 20), non-LDASA+NSAID (n = 25), and comparison (n = 12) groups, as well as their association with the presence or absence of* H. pylori*.

GM changes were significantly more often encountered in the NSAID+LDASA group (19/20) than in the comparison one (8/12) (*P = 0.05*; OR = 9.5; 95% CI, 0.91-98.81) and in the non-LDASA+NSAID group (16 (64.0%)/25) (*P = 0.014*; OR = 10.69; 95% CI, 1.22-93.64). There were no significant differences in the detection rate of GM changes in the non-NSAID+LDASA and comparison groups.

GM* H. pylori* was found in 13 (65.0%) of the 20 patients in the NSAID+LDASA group and did not differ significantly from the detection rate of* H. pylori* in the comparison and non-LDASA+NSAID groups. There were no significant differences in the detection of* H. pylori* in the presence of various GM changes between the NSAID+LDASA, non-LDASA+NSAID, and comparison groups (*P*_*2*_,* P*_*4*_ in Table 9).

Antral gastritis was observed significantly more often in the NSAID+LDASA group (16/20) than in the comparison one (5/12) (*P*_*1*_* = 0.034*; OR = 5.6; 95% CI, 1.15-27.37). There were no significant differences in the detection rate of antral gastritis in the NSAID+LDASA and non-LDASA+NSAID groups (Table 9).

The detection rates for pangastritis, GM erosions, and hemorrhages were statistically insignificant in the NSAID+LDASA, non-LDASA+NSAID, and comparison groups (P_1_ and P_3_, Table 9). There were no significant differences in the detection rate for pangastritis, GM erosions, and hemorrhages in the non-LDASA+NSAID and comparison groups.

## 5. Discussion

There are now sufficiently detailed reports on damage to various organs and systems in SLE and APS, which are caused by an underlying disease. GM changes may occur as a result of activity of the underlying disease, exposure to infectious agents, and ongoing drug therapy. Our endoscopic study has shown that 90.6% of patients with SLE and APS have GM changes that are commonly asymptomatic: only 10% of patients were active in complaining at the time of their examination. The symptoms of abdominal discomfort, including pains with or without nausea, as well as heartburn, nausea without pain syndrome are the most common complaints in both patients with SLE and those with APS. Our study has indicated that are no significant differences in SLEDAI scores between patients with and without gastrointestinal symptoms. However, the examined patients with GM damage had a higher SLEDAI score than those with intact GM (17 ± 4 and 16 ± 6).

Epigastric pain was detected in 37 (43.5%) of the 85 examined patients with SLE and APS. Nine (10.6%) of them had severe pain syndrome that required excluding acute surgical disease. In these patients, the pain was a manifestation of adhesive polyserositis and erosive gastritis in 3 and 6 cases, respectively. Pain was significantly more frequent complaint in APS patients in comparison to those with SLE. According to various authors, the incidence of abdominal pain in SLE patients ranges from 8 to 37% and the assessment of its cause is quite complex [35–41]. The more frequent detection of pain in our examined patients than that reported in the literature is most likely associated with the inclusion of APS patients in the study. The reasons why pain syndrome was more frequently detected in patients with APS may be associated with vascular changes [40, 42]. According to Fawzy et al., gastrointestinal manifestations were detected in 42.5% of SLE patients, while 6% of patients were found to have acute abdominal pain due to pleuritis or peritonitis in the presence of SLE activity [37]. Yuan et al. performed retrospective cohort study of SLE patients with complaint of abdominal pain. Lupus mesenteric vasculitis and lupus pancreatitis were the leading causes of SLE-induced abdominal pain [41].

Nausea and heartburn were detected in 22 (26%) of the 85 examined patients, which agreed with the data available in the literature. Vomiting, nausea, and anorexia in SLE are associated with severity and life-threatening conditions due to high SLE activity. However, the same manifestations may be related to drug therapy. In his review, D. Hallegua noted that the incidence of nausea in SLE patients ranged from 11 to 38% [35]. This figure dropped to 8% after excluding patients with nausea caused by medication.

Due to the fact that 62.3% of the patients presented no complaints at the time of examination, it is necessary to more actively and purposefully make inquiries about gastrointestinal complaints for the early detection of gastrointestinal signs in patients with SLE and APS.

Gastritis and peptic ulcer disease (PUD) remain the most common stomach changes in SLE, as reported by various authors [35–37]. Our endoscopic study revealed that SLE and APS patients had the following GM changes: antral gastritis, erosions, hemorrhages, and pangastritis. And only 9.4% of the examinees had intact GM. GM damages and the mechanism of their development in SLE and APS are unknown and continue to be discussed [9, 33, 35–37]. The identified GM changes in SLE and APS may be due to the course of disease, long-term, sometimes lifetime, drug intake, GM infection with* H. pylori*, or a set of these factors.

Over the past two decades, many researchers have discussed the possible role of* Herpesviridae* viruses, particularly* Herpes simplex types 1 and 2* (*HSV-1*,* HSV-2*) and* Cytomegalovirus* (*CMV*), in the development of peptic ulcers of the stomach and duodenum [43–45]. The viruses of the* Herpesviridae* family were identified in 46 (54.1%) of 85 examined patients with SLE and APS: these were found alone and concurrent with* H. pylori* in 10 and 36 patients, respectively. The detection rates of* HSV-1* and* CMV* were higher in patients with APS.* CMV* monoinfection in patients with SLE and APS was virtually unassociated with GM changes: antral gastritis was identified in only three* CMV* monoinfected patients. In our examined HSV-1 monoinfected patients, 7 were found to have antral gastritis that was associated with GM erosions in 2 patients and with GM hemorrhages in 2. At the same time, it is difficult to judge the effect of these infectious agents on the development of GM damages since coinfection with* H. pylori*+*HSV-1*+*CMV* was noted in 50% of cases among the patients with intact GM. It is known that the viruses of the* Herpesviridae* family in SLE patients can cause a number of serious complications, including necrotic ileitis [44]. The herpesviruses first colonize the mucosal epithelial cells, which may result in GM damage. The detection rate of specific antibodies against the viruses of the* Herpesviridae* family has been shown to be significantly higher in SLE patients than in the general population [46]. The frequency of GM changes in patients with coinfection (*H. pylori*+*HSV-1*+*CMV*) was comparable with the number of* H. pylori* monoinfected patients. The detection rate of GM* H. pylori* in SLE and APS patients was significantly higher than that of* HSV-1* and* CMV*. The found GM changes in the examined patients with SLE and APS were most likely to be unassociated with* HSV-1* and* CMV*. These data are in agreement with those given in the works suggesting that the* Herpesviridae* play no significant role in damaging the GM in SLE and APS [47–49]. Despite the fact that there are no statistically significant changes in the association of GM damage with* HSV-1*,* CMV* and with the activity of the disease itself, the mutually aggravating impact of these processes cannot be ignored.

Ramos-Casals M et al. analyzed the etiology and clinical features of acute viral infections in patients with SLE [26]. They included 88 cases (23 from their clinics and 65 from the literature review-MEDLINE searching for additional cases reported between January 1985 and March 2008) of acute viral infections in patients with SLE. The most common viral infections in patients with SLE were parvovirus B19 (predominantly mimicking SLE presentation) and CMV (predominantly presenting in severely immunosuppressed patients). CMV infection may mimic a lupus flare or present with specific organ involvement such as gastrointestinal bleeding or pulmonary infiltrates. Other herpesviruses are common in immunosuppressed SLE patients and may produce a wide range of manifestations. [26]. Another study noted a high seroprevalence of Human herpesvirus 8 infection was found in patients with SLE. The prevalence of Human herpesvirus 8 antibodies in SLE and normal controls was 57.8% (26/45) and 19.2% (5/26), respectively. These data were highly significant (P = 0.001) [50].

To date, the prevalence of* H. pylori* in SLE and APS patients and its significance in the development of GM damage have not been clearly determined. Some studies have shown that there is an association of* H. pylori* with various autoimmune diseases and that its seropositivity is related to the presence of antinuclear antibodies, anti-double-stranded DNA antibodies, and anti-Ro antibodies [51, 52]. The study conducted by Musaev et al. to compare GM histological data from 27 children with SLE and 12 with gastroduodenitis revealed the predominance of an inflammatory component in SLE, including the presence of fibroblasts and immunoglobulin deposition in the vessels of the GM microcirculation [53]. These findings were associated with SLE activity.

In our study,* H. pylori* was dominant among the investigated infectious agents associated with GM changes detected at EGD in patients with SLE and APS. The detection rate of* H. pylori* in the gastric biopsy specimens of the examined patients was high and amounted to 77.6%, which corresponds to its prevalence in the general population. The prevalence of* H. pylori* ranges from 35% to 90% in different populations [54–58]. It presents in 70%-90% of the population in developing countries and 35%-40% in developed ones [57, 59]. It is reported that 88% of the Moscow working population is infected with* H. pylori*. Its prevalence is 78% in people younger than 30 years and about 97% in individuals older than 60 years [60].

Along with* H. pylori*, drug therapy may be one of the possible causes of GM damage in patients with SLE and APS. Patients with these conditions are prescribed GCs, ACs, LDASA, NSAIDs, and antiplatelet drugs, as clinically indicated. In SLE and APS, these drugs are generally taken for a long time, sometimes during life, which can contribute to GM damage. It is customary to consider that therapy with GCs, ACs, LDASA, NSAIDs is associated with frequent adverse effects on the gastrointestinal mucosa. Despite the relevance of this issue, studies on the effect of the above drugs on GM in patients with SLE and APS remains poorly explored [7, 8, 19, 38, 40, 42].

Glucocorticoids (GCs) are widely used in clinical practice to treat systemic autoimmune diseases. Despite their important clinical efficacy, GCs produce several adverse reactions, most are time and dose dependent, limiting their clinical usefulness. These adverse effects are particularly relevant in chronic diseases that require long treatment periods [61]. Patients with SLE usually receive high-dose oral or pulse GC therapy (1000 mg/day for 2-3 days) to suppress disease activity. The dose of corticosteroids may be more than 100 times higher than the physiological blood concentration of GCs [61, 62]. The risk factor for steroid-induced gastric ulcers has been noted to be an intake of high-dose prednisolone for 30 days or more and evidence for PUD in the history in GC users [61, 63–65]. Inhibition of prostaglandin synthesis is one of the mechanisms of ulcerogenic action of exogenous GCs used in pharmacological doses [30]. According to our data, GCs administration in patients with SLE leads to a significant rise in the number of cases of endoscopic GM changes. At the same time, the longer the patients do not take GCs, the lower the detection rate of antral gastritis (88.6%>66.7%>41.7%) and GM erosions (27.3%>19.1%>16.7%) in SLE patients is(Table 6). And the detection rate of pangastritis and hemorrhages did not depend on the timing of GCs discontinuation. We did not analyze GC dose-dependent GM changes in the examined patients. But they all received GCs for more than 1 year [5.6 ± 7.2 years (1 to 33)].

The manifestations of SLE, such as serositis and musculoskeletal symptoms, are often treated with NSAIDs. Naproxen, salicylates, sulindac, and ibuprofen are the most frequently used agents [66]. In addition to the treatment of pain and inflammation, low-dose aspirin is used as a platelet and anticoagulant agent to treat APS [67, 68]. Few studies have evaluated the efficacy or the safety profile of NSAIDs in SLE patients and most National Drug Administrations have not approved them for this disease [19, 30, 66]. All NSAIDs at the doses effectively controlling inflammation carry a risk for serious gastrointestinal side effects, such as bleeding, ulcers, and perforations [19]. These complications are the leading cause of drug-related hospital admissions. Considering the fact that the gastrointestinal manifestations of SLE are found in up to 50% of patients, it is surprising that there is no report on the true incidence and prevalence of gastroduodenal ulcers in this disease.

The description of GM damages in SLE patients revealed that identified gastritis and PUD were largely associated with the administration of anti-inflammatory drugs and primarily NSAIDs [19, 30, 66, 69]. It is known from clinical observations that hormone therapy consisting of glucocorticoid hormones at pharmacological doses increases the risk of ulcerogenic disease induced by NSAIDs [30]. The myth that there are safer agents among NSAIDs has not been confirmed [66]. Griffin and Smalley have shown that there are no safe NSAIDs; all of them have all dose-related damaging effect on GM [70, 71]. Moreover, in their work, Griffin and Smalley refute the opinion that GM adaptation to NSAIDs can occur during their long-term use [70].

LDASA (75-32 mg/day) is usually prescribed for the prevention of cardiovascular and cerebrovascular events. Today, there are a growing number of patients taking LDASA for the prevention of vascular complications. Prospective and retrospective studies in Japan have shown that the rate of GM injury during long-term LDASA therapy ranges from 48 to 62% [72]. According to the data of these studies, LDASA therapy has two of five GM damage factors in any combination: age and coadministration of drugs, such as ACs, NSAIDs, GCs, and antiplatelet drugs. We did not carry out a multivariate analysis due to the small number of patients in the groups; however, our data suggest that GM damage in SLE and APS patients is more often observed during combination therapy with GCs, NSAIDs, LDASA, and ACs. A combination of drugs (GCs, NSAIDs, LDASA, and ACs) does not rule out the role of the disease itself in the damaging effect on GM.

A complex analysis of the effect of* H. pylori* and pathogenic therapy drugs on GM was carried out in patients with SLE and APS. GC, AC, LDASA, or NSAID users showed a high GM damage detection rate (90.6% of cases).

Comparison of the groups of patients with and without changes in the GM revealed that its damage was significantly more common in GC, AC, LDASA, or LDASA+NSAID users. Our findings are consistent with those reported by Griffin and Smalley [70, 71]. The authors have shown that the combination of NSAID, LDASA+NSAID, GCs, and ACs in any combination is an additional risk factor for GM damage. At the same time, our examined patients were found to have GM damages, such as antral gastritis, pangastritis, erosions, and hemorrhages, which were unassociated with* H. pylori*. Endoscopic and PCR data suggest that* H. pylori* plays no significant role in GM damage in SLE and APS, which agrees with the data available in the literature [14, 49, 73]. It can be said that in patients with SLE and APS, the GM is colonized by* H. pylori *[74]. At the same time, its value in GM damage in patients with SLE and APS has not been proven. Moreover, the literature contains information about the protective role of* H. pylori* in the development of SLE [51]. The authors have anticipated that* H. pylori* play a certain role in immunoregulatory events [51, 52]. There is evidence that* H. pylori* may confer benefits to humans [75]. There is a growing body of work suggesting that* H. pylori* may behave like a commensal or a symbiont, depending upon the circumstances [76–78]. The data on the potential importance of* H. pylori* to human health are discussed in the review article by Cover T. and Blaser M. [79]. Despite the fact that we have not found an association of GM damage with* H. pylori*, the role of the latter in the development of SLE and APS remains unclear, which requires further investigations. It is assumed also that clarifying the role of gastrointestinal communities in* H. pylori*-associated diseases will provide an opportunity for translational application as a biomarker for the risk of serious* H. pylori* diseases [80].

Analyzing the patients receiving and not receiving GCs, ACs, LDASA, and LDASA+NSAIDs showed that* H. pylori* was significantly more frequently detected in the GM of LDASA and ACs users. We also noted that among the patients with GM changes,* H. pylori* was significantly more common in LDASA and ACs users than that in GCs users. At the same time, in GC, LDASA+NSAID, AC, and LDASA users, the detection rate of* H. pylori* was comparable with its prevalence in the general population and amounted to 69.8, 68.4, 87.2, and 89.2%, respectively. The findings suggest that the administration of GCs and NSAIDs is accompanied by a decline in the amount of* H. pylori* in the GM of SLE and APS patients. Taking into account that these patients received no anti-*H. pylori* therapy at the time of their examination, the given data suggest that ACs and LDASA are most likely to have no effect on the level of GM colonization by* H. pylori*. This may be due to the fact that the patients took enteric-coated aspirin. This coating undoubtedly reduces the contact of the active ingredient of the drug with the GM and* H. pylori* [30, 81]. At the same time, the coadministration of LDASA and NSAIDs causes a decrease in the detection rate of* H. pylori* in patients with SLE and APS, which does not exclude the effect of NSAIDs on this bacterium. GCs, like NSAIDs, may have the same effect on* H. pylori*. ACs are most likely to be drugs that do not themselves affect* H. pylori*.

Analysis of GM changes in relation to the administration of a particular drug revealed that the development of antral gastritis in SLE and APS patients was significantly associated with the intake of GCs, ACs, LDASA, and NSAIDs. Accordingly, the use of GCs, ACs, and NSAIDs is accompanied by a significant decline in the number of patients with intact GM. And only the intake of LDASA does not result in a significant decrease in the number of patients with intact GM. Considering that* H. pylori* is present in approximately the same ratio in patients with intact GM and antral gastritis when using the drugs, it can be said that* H. pylori* does not play a significant role in GM damage in SLE and APS. This is confirmed by the data available in the literature. Thus, Luo et al. note that NSAID/aspirin rather than* H. pylori* infection increases GM damage in patients who receive methylprednisolone as pulse therapy [8].

The development of pangastritis was seen in SLE and APS patients taking GCs, ACs, and LDASA+NSAID and was not significantly associated with the administration of LDASA. Erosions and hemorrhages were more common in patients with APS and were most likely to have a complex mechanism associated with vascular disease, the presence of infectious agents, and drug therapy.

## 6. Conclusion

Endoscopic examination revealed that SLE and APS patients had the following GM damage: antral gastritis (82.4%), erosions (24.7%), hemorrhages (8.2%), and pangastritis (8.2%). In SLE and APS, the frequency of GM damage did not differ statistically. EGD detected significantly more frequently multiple GM erosions in patients with APS than in those with SLE without APS. It is apparent that this is due to GM microcirculatory bed pathology and, as a consequence, to the development of ischemia. The intact GM was present in 9.4% of patients with SLE and APS.

The found GM changes are most often clinically asymptomatic in patients SLE and APS. Only 10% of patients complain of epigastric discomfort. Endoscopic examination showed no correlation between GM damage and SLEDAI activity in patients with SLE and APS.

The gastric biopsy specimens were found to have infectious agents, such as* H. pylori*,* HSV-1*, and* CMV*, in 88.2% of the examined patients. None of the identified infections was present in only 11.8% of the gastric biopsy specimens.* H. pylori* was a dominant infectious agent in the GM. The concurrence of* H. pylori* with* Herpesviridae* in the gastric biopsy specimens was observed in 48.0% of patients; and* H. pylori* monoinfection was seen in 52.0%.* HSV-1* and* CMV* were identified in the gastric biopsy specimens from SLE and APS patients in 39% and 26% of cases, respectively. There was no evidence that* H. pylori*,* HSV-1*, and* CMV* play a role in GM damage in SLE and APS.

There was a high frequency of GM colonization by* H. pylori* in patients with SLE and APS. The rate of* H. pylori* infection in patients with SLE and APS corresponded to that in the general population. The group of patients with SLE and APS did not differ statistically in the frequency and degree of GM* H. pylori* colonization. There was no direct correlation between the found GM damage and the presence of* H. pylori* in SLE and APS patients. There were significant differences in the detection rate of* H. pylori* in relation to the drugs used: more frequent detection of* H. pylori* in AC and LDASA users than in GC and NSAID ones.

Drug therapy with GCs, LDASA, NSAIDs, and ACs was associated with the development of GM damage in patients with SLE and APS. Antral gastritis was significantly more often detected in the users of these drugs than in those who did not receive drug therapy for six months or more (a comparison group).

Our findings suggest that the found GM damage in SLE and APS have a complex multifactorial mechanism and require further investigation.

## Figures and Tables

**Table 1:** Characteristics of patients included in the study.

Parameters	Value of parameters in the groups		
	PAPS,	SLE + APS,	SLE,
	n = 20	n = 26	n = 39
Mean age, year ± SD	34.0 ± 10.8	40.0 ± 11.8	35.5 ± 13.1

Disease duration, year ± SD	8.1 ± 7.3	16.0 ± 11.6*∗∗*	10.2 ± 10.0

Females/males, n (%)	12 (60)/8 (40)	22 (85)/4 (15)	36 (92)/3 (8)

Disease activity, SLEDAI-index (mean scores ± SD)	-	19.4 ± 8.6	20.0 ± 12.1

Thrombosis in past history, n (%)	16 (80)	18 (69)	-

Obstetrical morbidity, n (%)*∗*	5 (20)	11 (42.3)	-

Therapy at the time of study inclusion			

GCs, n (%)	0	23 (89)	21 (54)

Anticoagulants (LMWH, VKA), n (%)	20 (100)	26 (100)	3 (7.7)

LDASA, n (%)	20 (100)	26 (100)	14 (36)

NSAID, n (%)	1 (5)	9 (34.6)	10 (25.6)

Patients [n (%)] who did not receive GCs, ACs, LDASA, NSAID+LDASA (comparison group) during 6 months or more	-	-	12 (30.8)

Note: SD: standard deviations; GCs: glucocorticoids; LDASA: low-dose acetylsalicylic acid; NSAID: nonsteroidal anti-inflammatory drugs; ACs: anticoagulants; LMWH: low-molecular-weight heparin; VKA: vitamin K antagonist.

*∗* The percentage was calculated from the number of women who had a pregnancy during the disease.

*∗∗*P < 0.005 compared to those in PAPS patients.

**Table 2:** The detection rate of *H. pylori, HSV-1, CMV* in the gastric mucosa of patients with SLE and APS.

Infectious agents	The detection rate of infectious agents in the groups			
	PAPS, n = 20	SLE+APS, n = 26	SLE, n-39	All patients, n=85
*H. pylori*, n (%)	14 (70.0)	21 (80.8)	31 (79.5)	66 (77.6)*∗*#
*CagA,* n (%)	9 (45.0)	13 (50.0)	25 (64.1)	47 (55.3)
*HSV-1,* n (%)	11 (55.0)	10 (38.5)	12 (30.8)	33 (38.8)*∗*
*CMV,* n (%)	6 (30.0)	6 (23.1)	10 (25.6)	22 (25.9)#
*No infections*, n (%)	4 (20.0)	3 (11.5)	3 (7.7)	10 (11.8)

*Note*:

*∗*- *χ*^2^ = 26.18; P = 0.00003; OR = 5.47; at 95% CI, RR = 2.0<1.50<RR<2.67; comparison of the detection rate for *H. pylori *and* HSV-1.*

*#*- *χ*^2^ = 45.34; P < 0.0001; OR = 9.95; at 95% CI, RR = 3.00<2.06<RR<4.38; comparison of the detection rate for *H. pylori *and* CMV*.

**Table 3:** Detection rates of *Helicobacter pylori*, *HSV-1*, *CMV*, and their combinations in various gastric mucosal damages, as evidenced by esophagogastroduodenoscopy.

Gastric mucosa status	*H. pylori *	*HSV-1*	*CMV *	*H.pylori + HSV-1 + CMV *
	(n = 29)	(n = 7)	(n = 3)	(n = 36)
Antral gastritis,* n (*%)	26 (89.7)	7 (100.0)	3 (100.0)	28 (77.8)
Pangastritis,* n (*%)	4 (13.8)	0	0	3 (8.3)
Gastric mucosal erosion,* n (*%)	8 (27.6)	2 (28.6)	0	9 (25.0)
Gastric mucosal hemorrhage,* n (*%)	4 (13.8)	2 (28.6)	0	1 (2.8)
Intact gastric mucosa,* n (*%)	1 (3.4)	1 (14.3)	0	4 (11.1)

*Note*: The percentage of positive results was calculated from the number of patients having an infectious agent.

**Table 4:** Endoscopic gastric mucosal changes in the examined patients and detection rates of *H. pylori*.

Gastric mucosa status at endoscopy	PAPS		SLE+APS		SLE		All patients	
	n = 20		n = 26		n = 39		n = 85	
	*H. pylori* (+)	*H. pylori* (-)	*H. pylori* (+)	*H. pylori* (-)	*H. pylori* (+)	*H. pylori* (-)	*H. pylori* (+)	*H. pylori* (-)
	n = 14 (70.0%)	n = 6 (30.0%)	n = 21 (80.8%)	n = 5 (19.2%)	n = 31 (79.5%)	n = 8 (20.5%)	n = 66 (77.6%)	n = 19 (22.4%)
All patients with gastric mucosal changes, n (%)	12 (85.7)	6 (100)	21 (100)	5 (100)	28 (90.3)	5 (62.5)	61 (92.4)	16 (84.2)

Antral gastritis, n (%)	11 (78.6)	6 (100)	20 (95.2)	5 (100)	23 (74.2)	5 (62.5)	54 (81.8)	16 (84.2)

Pangastritis, n (%)	1 (7.1)	0	1 (4.8)	0	5 (16.6)	0	7 (10.6)	0

Gastric mucosal erosion, n (%)	5 (35.7)	0	7 (33.3)	3 (60.0)	5 (16.6)	1 (12.5)	17 (25.6)	4 (21,2)

Gastric mucosal hemorrhage, n (%)	1 (7.1)	1 (16.7)	2 (9.5)	1 (20.0)	2 (6.4)	0	5 (7.6)	2 (10.5)

Intact gastric mucosa, n (%)	2 (14.3)	0	0	0	3 (10.0)	3 (33.3)	5 (7.8)	3 (14.3)

*Note*: *H. pylori *was detected by PCR.

**Table 5:** Detection rates for GM injuries and *H. pylori* according to the administration of drugs.

Patient groups receivingdrugs at the time of study inclusion, n = 73							
Drugs	*H. pylori*	Altered gastric mucosa,		Intact gastric mucosa,		P; OR; 95% CI	
		n = 69		n = 4			

Glucocorticoids, n (%)	(+)	43 (62.3)	30 (69.8)	1 (100.0)	1 (25.0)	*P*_*1*_ * = 0.0059*; OR = 21.50; 95% CI 2.12-218.27	P_2_ = 0.70; OR = 0; 95% CI 0-NaN
	(-)		13 (30.3)	0			

ACs, n (%)	(+)	47 (68.1)	41 (87.2)	1 (50.0)	2 (50.0)	*P*_*1*_ * = 0.011*; OR = 11.75; 95% CI 1.84-75.15	P_2_ = 0.27; OR = 6.83; 95% CI 0.38-124.34
	(-)		6 (12.8)	1 (50.0)			

LDASA, n (%)	(+)	37 (53.6)	33 (89.2)	2 (66.7)	3 (75.0)	*P*_*1*_ * = 0.041*; OR = 6.17; 95% CI 1.15-33.11	P_2_ = 0.34; OR = 4.13; 95% CI 0.30-56.39
	(-)		4 (10.8)	1 (33.3)			

NSAID+LDASA, n (%)	(+)	19 (27.5)	13 (68.4)	0	1 (25.0)	*P*_*1*_ * = 0.05*; OR = 9.5; 95% CI 0.91-98.81	P_2_ = 0.35; OR = 0 95% CI 0
	(-)		6 (31.6)	1 (100.0)			

Patient group not receiving GCs, ACs, LDASA, NSAID + aspirin for 6 months or more (comparison group); n = 12							

No drugs, n (%)	(+)	8 (66.7)	5 (62.5)	2 (50.0)	4 (33.3)	*P*_*1*_	-
	(-)		3 (37.5)	2 (50.0)			

*Notes:* LDASA – low-dose acetylsalicylic acid; NSAID – nonsteroidal anti-inflammatory drugs; ACs – anticoagulants. The percentage of patients with altered or intact GM was calculated from the number of all the patients in group.

The percentage of *H. pylori*-positive patients was calculated from the number of those in the group receiving the appropriate drug.

P_1_ - comparison of the number of patients with and without GM changes who took the appropriate drug with that in the comparison group.

P_2_ - comparison of the number of patients taking the appropriate drug and having and not having GM changes according to the presence or absence of *H. pylori*.

**Table 6:** Gastric mucosal changes at EGD in the patient groups according to the administration of glucocorticoids and the rate of *H. pylori* detection.

Altered gastric mucosa	*H. pylori*	Patients receiving glucocorticoids,		Patients not receiving glucocorticoids,		Comparison group,		P; OR; (95%CI)			
		n = 44		n = 21		n = 12					
Antral gastritis, n (%)	(+)	31 (85.9)	39 (88.6)	10 (71.4)	14 (66.7)	3 (60.0)	5 (41.7)	*P*_*1*_ *= 0.0017*; OR = 10.92 (95% CI 2.49-47.87)	P_2_ = 0.32; OR = 2.58 (95% CI 0.37-18.17)	*P*_*3*_ *= 0.039*; OR = 3.9 (95% CI 1.06-14.31)	P_4_ = 0.39; OR = 1.55 (95% CI 0.38-6.26)
	(-)	8 (14.1)		4 (28.6)		2 (40.0)					

Pangastritis, n (%)	(+)	4 (100.0)	4 (9.1)	2 (100.0)	2 (9.5)	2 (100.0)	2 (16.7)	P_1_ = 0.38; OR = 0.5 (95% CI 0.08-3.13)	P_2_ = 1	P_3_=0.64; OR=0.95 (95% CI 0.16-5.65)	P_4_ = 1
	(-)	0		0		0					

Gastric mucosal erosion, n (%)	(+)	9 (75.0)	12 (27.3)	3 (75.0)	4 (19.1)	2 (100.0)	2 (16.7)	P_1_ = 0.37; OR = 1.88 (95% CI 0.36-9.83)	P_2_ = 0.60; OR = 0 (95% CI 0-NaN)	P_3_ = 0.27; OR=1.82 (95% CI 0.51-6.57)	P_4_ = 0.73; OR = 1 (95% CI 0.07-13.64)
	(-)	3 (25.0)		1 (25.0)		0					

Gastric mucosal hemorrhage, n (%)	(+)	3 (100.0)	3 (6.8)	1 (50.0)	2 (9.5)	1 (100.0)	1 (8.3)	P_1_ = 0.63; OR = 0.80 (95% CI 0.08-8.52)	P_2_ = 1	P_3_=0.52; OR=0.70 (95% CI 0.11-4.51)	P_4_ = 0.40 OR = Infinity (95% CI NaN-Infinity)
	(-)	0		1 (50.0)		0					

Intact gastric mucosa, n (%)	(+)	1 (100.0)	1 (2.3)	2 (40.0)	5 (23.8)	2 (50.0)	4 (33.3)	*P*_*1*_ * = 0.006*; OR = 21.5 (95%CI 2.12-218.27)	P_2_ = 0.6; OR = Infinity (95% CI NaN-Infinity)	*P*_*3*_ * = 0.011*; OR = 0.07 (95% CI 0.008-0.69)	P_4_ = 0.5; OR=Infinity (95% CI NaN-Infinity)
	(-)	0		3 (60.0)		2 (50.0)					

*Notes*: The percentage of patients with appropriate GM changes in the glucocorticoid, non-glucocorticoid, and comparison groups was calculated from the total number of patients in the corresponding group.

The percentage of *H. pylori*-positive patients was calculated from the number of those with appropriate GM changes in the glucocorticoid, non-glucocorticoid, and comparison groups.

P_1_ - comparison of the number of patients with and without appropriate GM changes in the comparison and glucocorticoid groups.

P_2_ - comparison of the number of *H. pylori*-positive and *H. pylori*-negative patients with an appropriate GM change in the comparison and glucocorticoid groups.

P_3_ - comparison of the number of patients with and without appropriate GM changes in the glucocorticoid and non-glucocorticoid groups.

P_4_ - comparison of the number of *H. pylori*-positive and *H. pylori*-negative patients with an appropriate GM change in the glucocorticoid and non-glucocorticoid groups.

**Table 7:** Gastric mucosal changes at EGD according the administration of anticoagulants and the rate of *H. pylori* detection.

Altered gastric mucosa	*H. pylori*	Patients receiving anticoagulants,		Patients not receiving anticoagulants,		Comparison group,		P; OR (95%CI)			
		n = 49		n = 36		n = 12					
Antral gastritis, n (%)	(+)	40 (88.9)	45 (91.8)	16 (72.7)	22 (61.1)	3 (60.0)	5 (41.7)	*P*_*1*_ * = 0.0004*; OR = 15.75 (95% CI 3.39-73.26)	P_2_ = 0.14; OR = 5.33 (95% CI 0.71-40.06)	*P*_*3*_ * = 0.00076*; OR = 7.16, (95% CI 2.11-24.31)	P_4_ = 0.09; OR = 3 (95% CI 0.80-11.24)
	(-)	5 (11.1)		6 (27.3)		2 (40.0)					

Pangastritis, n (%)	(+)	2 (100.0)	2 (4.1)	5 (100.0)	5 (13.9)	2 (100.0)	2 (16.7)	P_1_ = 0.17; OR = 0.21 (95% CI 0.03-1.70)	P_2_ = 1	P_3_ = 0.11; OR = 0.26 (95% CI 0.05-1.45)	P_4_ = 1
	(-)	0		0		0					

Gastric mucosal erosion, n (%)	(+)	10 (90.9)	11 (22.4)	8 (80.0)	10 (27.8)	2 (100.0)	2 (16.7)	P_1_ = 0.50; OR = 1.45 (95% CI 0.28-7.61)	P_2_ = 0.85; OR = 0 (95% CI 0-NaN)	P_3_ = 0.38; OR = 0.75 (95% CI 0.28-2.03)	P_4_ = 0.46; OR = 2.5 (95% CI 0.19-32.80)
	(-)	1 (9.1)		2 (20.0)		0					

Gastric mucosal hemorrhage, n (%)	(+)	4 (100.0)	4 (8.2)	2 (66.7)	3 (8.3)	1 (100.0)	1 (8.3)	P_1_ = 0.68; OR = 0.98 (95% CI 0.10-9.64)	P_2_ = 1	P_3_ = 0.64; OR = 0.98 (95% CI 0.20-4.67)	P_4_ = 0.43; OR = Infinity (95% CI NaN-Infinity)
	(-)	0		1 (33.3)		0					

Intact gastric mucosa, n (%)	(+)	2 (100.0)	2 (4.1)	4 (66.7)	6 (16.7)	2 (50.0)	4 (33.3)	*P*_*1*_ * = 0.011*; OR = 11.75; (95% CI 1.84-75.15)	P_2_ = 0.4; OR = Infinity (95% CI NaN-Infinity)	P_3_ = 0.057; OR = 0.21 (95% CI 0.04-1.12)	P_4_ = 0.54; OR = Infinity (95% CI NaN-Infinity)
	(-)	0		2 (33.3)		2 (50.0)					

*Notes*: The percentage of patients with appropriate GM changes in the anticoagulant, non-anticoagulant, and comparison groups was calculated from the total number of patients in the corresponding group.

The percentage of *H. pylori*-positive patients was calculated from the number of those with appropriate GM changes in the anticoagulant, non-anticoagulant, and comparison groups.

Р_1_: comparison of the number of patients with and without appropriate GM changes in the comparison and anticoagulant groups.

Р_2_: comparison of the number of *H. pylori*-positive and *H. pylori*-negative patients with an appropriate GM change in the comparison and anticoagulant groups.

Р_3_: comparison of the number of patients with and without appropriate GM changes in the anticoagulant and non-anticoagulant groups.

Р_4_: comparison of the number of *H. pylori*-positive and *H. pylori*-negative patients with an appropriate GM change in the anticoagulant and non-anticoagulant groups.

**Table 8:** Gastric mucosal changes at EGD according to the administration of LDASA and the rate of *H. pylori* detection.

Altered gastric mucosa	*H. pylori*	Patients receiving LDASA,		Patients not receiving NSAID+LDASA,		Comparison group,		P; OR; (95%CI)			
		n = 40		n = 25		n = 12					
Antral gastritis, n (%)	(+)	27 (84.4)	32 (80.0)	14 (70.0)	20 (80.0)	3 (60.0)	5 (41.7)	*P*_*1*_ * = 0.016*; OR = 5.6 (95% CI 1.40-22.36)	P_2_ = 0.23; OR = 3.6 (95% CI 0.47-27.35)	P_3_ = 0.63; OR = 1.0 (95% CI 0.29-3.49)	P_4_ = 0.19; OR = 2.31 (95% CI 0.60- 8.94)
	(-)	5 (15.6)		6 (30.0)		2 (40.0)					

Pangastritis, n (%)	(+)	0	0	5 (100.0)	5 (20.0)	2 (100.0)	2 (16.7)	*P*_*1*_ * = 0.05*; OR = 0 (95% CI 0-NaN)	P_2_ = 1	*P*_*3*_ * = 0.006*; OR = 0 (95% CI 0-NaN)	P_4_ = 1
	(-)	0		0		0					

Gastric mucosal erosion, n (%)	(+)	10 (90.9)	11 (27.5)	3 (60.0)	5 (20.0)	2 (100.0)	2 (16.7)	P_1_ = 0.36; OR = 1.90 (95% CI 0.36-10.07)	P_2_ = 0.85; OR = 0 (95% CI 0 -NaN)	P_3_ = 0.35; OR = 1.52 (95% CI 0.46-5.04)	P_4_ = 0.21; OR = 6.67 (95% CI 0.44-101.74)
	(-)	1 (9.1)		2 (40.0)		0					

Gastric mucosal hemorrhage, n (%)	(+)	3 (75.0)	4 (10.0)	2 (100)	2 (8.0)	1 (100.0)	1 (8.3)	P_1_ = 0.68; OR = 1.22 (95% CI 0.12-12.11)	P_2_ = 0.80; OR = 0 (95% CI 0-NaN)	P_3_ = 0.58; OR = 1.28 (95% CI 0.22-7.55)	P_4_ = 0.67; OR = 0 (95% CI 0-NaN)
	(-)	1 (25.0)		0		0					

Intact gastric mucosa, n (%)	(+)	4 (80.0)	5 (12.5)	1 (50.0)	2 (8.0)	2 (50.0)	4 (33.3)	P_1_ = 0.11; OR = 3.5 (95% CI 0.76-16.05)	P_2_ = 0.40; OR = 4 (95% CI 0.21-75.66)	P_3_ = 0.45; OR = 0.61 (95% CI 0.11-3.41)	P_4_ = 0.52; OR = 4 (95% CI 0.12-136.97)
	(-)	1 (20.0)		1 (50.0)		2 (50.0)					

Notes: LDASA: low-dose acetylsalicylic acid.

The percentage of patients with appropriate GM changes in the LDASA, non-NSAID+LDASA, and comparison groups was calculated from the total number of patients in the corresponding group.

The percentage of *H. pylori*-positive patients was calculated from the number of those with appropriate GM changes in the LDASA, non- NSAID+LDASA, and comparison groups.

Р_1_: comparison of the number of patients with and without appropriate GM changes in the comparison and LDASA groups.

Р_2_: comparison of the number of *H. pylori*-positive and *H. pylori*-negative patients with an appropriate GM change in the comparison and LDASA groups.

Р_3_: comparison of the number of patients with and without appropriate GM changes in the LDASA and non-LDASA groups.

Р_4_: comparison of the number of *H. pylori*-positive and *H. pylori*-negative patients with an appropriate GM change in the LDASA and non-LDASA groups.

**Table 9:** Gastric mucosal changes at EGD according to the administration of NSAID+LDASA and the rate of *H. pylori* detection.

Altered gastric mucosa	*H. pylori*	Patients receiving NSAID+LDASA		Patients not receiving NSAID+LDASA,		Comparison group,		P; OR; (95% CI)			
		n = 20		n = 25		n = 12					
Antral gastritis, n (%)	(+)	11 (68.8)	16 (80.0)	14 (70.0)	20 (80.0)	3 (60.0)	5 (41.7)	*P*_*1*_ * = 0.034*; OR = 5.6 (95% CI 1.15-27.37)	P_2_ = 0.56; OR = 1.47 (95% CI 0.18-11.72)	P_3_ = 0.64; OR = 1 (95% CI 0.23-4.35)	P_4_ = 0.61; OR = 0.94 (95% CI 0.23-3.92)
	(-)	5 (31.2)		6 (30.0)		2 (40.0)					

Pangastritis, n (%)	(+)	2 (100.0)	2 (10.0)	5 (100.0)	5 (20.0)	2 (100.0)	2 (16.7)	P_1_ = 0.48; OR = 0.56 (95% CI 0.07-4.57)	P_2_ = 1	P_3_ = 0.31; OR = 0.44 (95% CI 0.08-2.58)	P_4_ = 1
	(-)	0		0		0					

Gastric mucosal erosion, n (%)	(+)	4 (80.0)	5 (25.0)	3 (60.0)	5 (20.0)	2 (100.0)	2 (16.7)	P_1_ = 0.47; OR = 1.67 (95% CI 0.27-10.33)	P_2_ = 0.71; OR = 0 (95% CI 0-NaN)	P_3_ = 0.48; OR = 1.33 (95% CI 0.33-5.45)	P_4_ = 0.50; OR = 2.67 (95% CI 0.16-45.14)
	(-)	1 (20.0)		2 (40.0)		0					

Gastric mucosal hemorrhage, n (%)	(+)	0	1 (5.0)	2 (100)	2 (8.0)	1 (100.0)	1 (8.3)	P_1_ = 0.62; OR = 0.58 (95% CI 0.03-10.21)	P_2_ = 0.5; OR = 0 (95% CI 0-NaN)	P_3_ = 0.58; OR = 0.61 (95% CI 0.05-7.20)	P_4_ = 0.33; OR = 0 (95% CI 0-NaN)
	(-)	1 (100.0)		0		0					

Intact gastric mucosa, n (%)	(+)	0	1 (5.0)	1 (50.0)	2 (8.0)	2 (50.0)	4 (33.3)	*P*_*1*_ * = 0.05*; OR = = 9.5; (95% CI 0.91-98.81)	P_2_ = 0.60; OR = 0 (95% CI 0-NaN)	P_3_ = 0.58; OR = 0.61 (95% CI 0.05-7.20)	P_4_ = 0.67; OR = 0 (95% CI 0-NaN)
	(-)	1 (100.0)		1 (50.0)		2 (50.0)					

*Notes*: The percentage of patients with appropriate GM changes in the NSAID+LDASA, non-NSAID+LDASA, and comparison groups was calculated from the total number of patients in the corresponding group.

The percentage of *H. pylori*-positive patients was calculated from the number of those with appropriate GM changes in the NSAID+LDASA, non- NSAID+LDASA, and comparison groups.

Р_1_ –comparison of the number of patients with and without appropriate GM changes in the comparison and NSAID+LDASA groups.

Р_2_ – comparison of the number of *H. pylori*-positive and *H. pylori*-negative patients with an appropriate GM change in the comparison and NSAID+LDASA groups.

Р_3_ – comparison of the number of patients with and without appropriate GM changes in the NSAID+LDASA and non-NSAID groups.

Р_4_ – comparison of the number of *H. pylori*-positive and *H. pylori*-negative patients with an appropriate GM change in the NSAID+LDASA and non- NSAID groups.

## Data Availability

All the data of our manuscript used to support the findings of this study are included within the article.
